# How Does District Health Management Emerge Within a Complex Health System? Insights for Capacity Strengthening in Ghana

**DOI:** 10.3389/fpubh.2020.00270

**Published:** 2020-07-08

**Authors:** Anne Christine Stender Heerdegen, Jana Gerold, Samuel Amon, Samuel Agyei Agyemang, Moses Aikins, Kaspar Wyss

**Affiliations:** ^1^Swiss Tropical and Public Health Institute, Basel, Switzerland; ^2^University of Basel, Basel, Switzerland; ^3^School of Public Health, College of Health Sciences, University of Ghana, Accra, Ghana

**Keywords:** management and leadership, capacity strengthening, district health systems, systems thinking, complex adaptive systems, low- and middle-income countries, Ghana

## Abstract

**Introduction:** District health managers (DHMs) play a pivotal role in the operation of district health systems in low—and middle income countries, including Ghana. Their capacity is determined by their competencies, but also by the organization and system in which they are embedded. The objective of this paper is to explore how district health management emerges from contextual, organizational, and individual factors in order to demonstrate that capacity strengthening efforts at district level need to transcend individual competencies to take on more systemic approaches.

**Methods:** Semi-structured interviews (*n* = 21) were conducted to gain insight into aspects that affect district health management in the Eastern Region of Ghana. Interviews were conducted with DHMs (*n* = 15) from six different districts, as well as with their superiors at the regional level (*n* = 4) and peers from non-governmental organizations (*n* = 2). A thematic analysis was conducted by using an analytical approach based on systems theory.

**Results:** Contextual aspects, such as priorities among elected officials, poor infrastructure and working conditions, centralized decision-making, delayed disbursement of funds and staff shortages, affect organizational processes and the way DHMs carry out their role. Enabling organizational aspects that provide DHMs with direction and a clear perception of their role, include positive team dynamics, good relations with supervisors, job descriptions, appraisals, information systems, policies and guidelines. Meanwhile, hierarchical organizational structures, agendas driven by vertical programs and limited opportunities for professional development provide DHMs with limited authority to make decisions and dampens their motivation. The DHMs ability to carry out their role effectively depends on their perception of their role and the effort they expend, in addition to their competencies. In regards to competencies, a need for more general management and leadership skills were called for by DHMs as well as by their superiors and peers.

**Conclusion:** Systemic approaches are called for in order to strengthen district health management capacity. This study can provide national policy-makers, donors and researchers with a deeper understanding of factors that should be taken into consideration when developing, planning, implementing, and assessing capacity-building strategies targeted at strengthening district health management.

## Introduction

In many low and middle-income countries (LMICs), including Ghana, district health managers' (DHMs) are the ones who facilitate the management and implementation of primary healthcare. They have to translate national health policies, as well as human, material and financial inputs, into accessible, high quality health services, and thus play a pivotal role in district health systems (1–4). Shortcomings within district health systems in LMICs, such as an unequally distributed health workforce, high levels of absenteeism, medicine stock-outs and poor health outcomes, are often attributed to weak management capacity (1, 3–9).

Capacity has been defined as the “*ability of individuals, organizations or systems to perform appropriate functions effectively, efficiently and sustainably*” (10). However, capacity strengthening within the health sector often focuses on enhancing the skills and technical capacity of individuals (11, 12). Individuals do however not operate in a vacuum, and their capacity is strongly influenced by the system and context in which they are embedded (12, 13).

Since the 1980s, management strengthening interventions (MSIs) have had a predominant focus on strengthening the DHMs' individual competencies rather than the system capacity (13–16). Individual competencies among DHMs are indeed critical for them to carry out their job in an effective and efficient way (3, 13, 14, 17–25). Nevertheless, it has been posed that leadership and management within complex adaptive systems, such as the district health system, need to be considered as more than the act of individuals, and rather as something that emerges through an interplay of many interacting forces (26). District health managers' operate within a context that incentivizes them to act in a certain way, and their practices are to a high extent influenced by and interdependent with other entities at the sub-district, district, regional and national level (3, 13, 14, 27–31).

Inadequate attention has been paid to the influence of the context and organizational structures in which DHMs operate in sub-Saharan Africa, including Ghana (13, 32). Thus, the aim of this paper is to explore how district health management emerges, hereunder the contextual, organizational and individual aspects that enable or hinder DHMs in carrying out their functions. The findings may provide national policy-makers, donors and researchers with a deeper understanding of factors that should be taken into consideration when developing, planning, implementing and assessing capacity-building strategies targeted at strengthening district health management. Moreover, findings may elicit the need to expand management strengthening beyond the traditional approaches that perceive managers as being outside the system with objective abilities to change the system (13).

### Management Structures Within the Ghana Health System

In Ghana, the Ministry of Health (MoH) are in charge of policy making and setting the strategic direction for the health sector. Meanwhile, the semi-autonomous agency Ghana Health Service (GHS) has been mandated by the MoH to implement the national health policies through management and operation of nearly all public health facilities. To take on this mandate, the GHS is administratively organized at the national, regional and district level (33). The GHS headquarters oversee the GHS Regional Health Administrations (RHA) that are located within each region. At the time this study took place, there were 10 regions in Ghana, subdivided into 216 districts. The RHAs, led by the Regional Director of Health Services, oversee the District Health Administrations (DHA) that are established within each district. The DHAs are run by district health management teams (DHMTs), who are responsible for the operation and management of public health facilities within their district, including health centers and Community Health and Planning Services (CHPS) (33). The DHMTs may consist of up to 12 core members that are led by a District Director of Health Services (DDHS). To our knowledge, there is no official document outlining core members of DHMTs, however they typically include administrative officers (i.e., DDHS, Deputy Director of Nursing Services and Health Administrators), technical officers (i.e., Public Health Nurse, Disease Control, Health Information, Nutrition, Health Promotion Officers), and operational officers (i.e., Finance, Human Resource and Supply Officers). The core managers are assisted by various program heads, for example coordinators of community health - and disease control programs (i.e., CHPS, Tuberculosis, Malaria and HIV coordinators). The DHMTs are vertically accountable to RHAs, who in turn are accountable to GHS headquarters. Moreover, the DHAs are horizontally accountable to the local governments, namely the district assemblies. The district assemblies are the highest political decision-making bodies within the districts, and play an important role in deciding how state resources are allocated within the districts (34). The funds allocated to health by the district assemblies in each district depends on the priorities of the district assembly and the lobbying power of the district health directorate (15). The private sector, non-governmental and faith-based organizations, as well as donor partners, also play an important role in adding resources and addressing challenges related to service delivery within the Ghana health system.

National initiatives to strengthen management and leadership at district level have taken place in Ghana, including the Leadership Development Program (LDP) and the Strengthening District Health System Initiative (SDHI) (13). The LDP has been implemented in districts across Ghana since 2008. It takes on a team-based approach in which DHMTs apply management and leadership practices (i.e., root cause analysis, action planning, monitoring and evaluation etc.) to address service delivery problems. Improved practices, team - and work climate were observed during the program and shortly thereafter, however were not sustained (3). The unsustainability was partially attributed to it being introduced in a top-down manner by regional officers, which diminished the DHMs own decision-making and thus ownership (3). The SDHI was implemented in the late 1980's. This program also focused on individual and team competencies, including problem analysis and problem solving (35). It was sustained for a while due to its focus on local ownership and a close-knit network between the then district leaders who shared management strengthening ideas amongst each other after the duration of the program. As a result of the program, the DHMTs became better planners and advocates for their needs as their capacity and confidence increased. Consequently, their decision-space was increased. Nonetheless, the momentum of the SDHI waned after a couple of years partly due to the existing district leaders no longer being in the districts, partly due to lack of financial support (35).

## Methods

### Study Setting

This study was conducted in six districts in the Eastern Region of Ghana. The districts were selected based on their involvement in the PERFORM2Scale project, which aims to scale-up a MSI at district level in Uganda, Malawi, and Ghana (www.perform2scale.org).

### Study Design and Population

This study took on an exploratory approach using qualitative interviews to gain insight into aspects that enable or hinder DHMs in carrying out their duties in a way that improves health service delivery and population health outcomes at district level. The DHMs were thus the core unit of research, however to verify their observations, a data source triangulation approach was applied (36). In addition to inviting DHMs to participate in the study, regional health administrators and staff from local non-governmental organizations (NGOs) within the study districts were invited to participate. The regional health administrators supervise the DHMTs and thus have a good oversight of the resources and support systems available at district level, as well as the individual capacity among DHMs. Meanwhile, the NGOs collaborate closely with the DHMTs in the field and have insights into how contextual, organizational or individual aspects affect the DHMs. Access to study participants were obtained through their involvement in the PERFORM2Scale project. The sampling strategy was purposive, as described by Ritchie et al. (37), however some of the invited DHMs could not participate due to a national mass distribution of long lasting insecticide-treated bed nets taking place at the same time as this study.

### Data Collection

Semi-structured in-depth interviews were carried out in February and March 2018, and were conducted by the first, third and fourth author of this paper. The interviews were facilitated by semi-structured interview guides, which were conceptualized based on the World Health Organizations leadership and management strengthening framework (6). Separate interview guides were developed for the DHMs, regional health administrators and the NGO staff, respectively. However, the three guides included similar questions relating to: (1) DHMs' roles and responsibilities; (2) DHMs qualifications; (3) required and perceived management competencies among DHMs; (4) DHMs relationship with external partners (NGOs, donors, academic institutions) and stakeholders at the national, regional, and sub-district level; and (5) the organizational and environmental context surrounding DHMs, and how it affects the DHMs in carrying out their responsibilities. Individual interviews were conducted at the study participants' workplace, and lasted ~40 minutes. All interviewees were informed about the interviewers' affiliation and the procedure of data collection.

### Data Analysis

**Figure 1:**
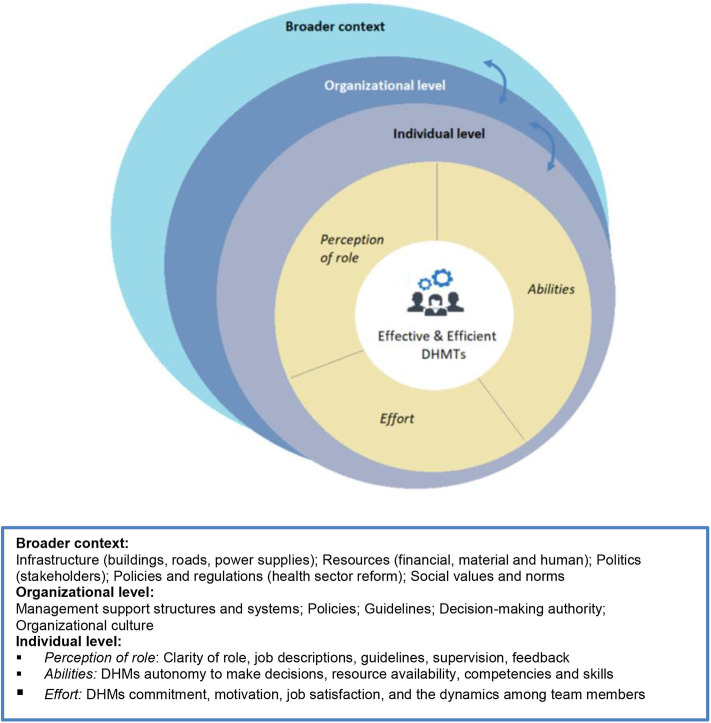
Framework on aspects affecting effective and efficient District Health Management.

The interviews were transcribed and subsequently coded in the qualitative research software Nvivo 12 by using a general inductive approach, as described by Hsieh and Shannon (38). Following a content analysis, global themes were generated by using a systems theory approach, in which data from DHMs, regional administrators and NGOs were organized into individual, organizational and contextual aspects affecting management capacity at district level (39–41) (Figure 1). The framework in Figure 1 demonstrates that the broader context refers to situational circumstances and characteristics that influence the behavior among DHMs, such as available resources, relationships with stakeholders, policies, and regulations. Meanwhile, the organizational context refers to the characteristics of the organization of GHS in which the DHMTs are embedded, including organizational processes and culture, available management support systems and structures, including the DHMs decision-making authority. Lastly, the individual aspects refer to (1) the DHMs perception of their role, (2) their abilities and (3) the efforts they put into carrying out their duties in an efficient and effective way. These three sub-themes have been described by Byars and Rue as affecting the degree to which an individual is fulfilling his or her assigned job tasks (42).

### Ethics

This study was carried out as an integral part of PERFORM2Scale under the lead of the Liverpool School of Tropical Medicine (LSTM). Ethical clearance was obtained from the Research Ethics Committee of LSTM (ID No. 17–046) and the GHS Ethics Review Committee (No. GHS-ERC004/01/20). Written informed consent was obtained from all study participants and they were informed about the possibility to withdraw from the interview at any time.

## Results

A total of 21 key stakeholders participated in the study, including fifteen DHMs, four regional health administrators and two staff members from two different health-related NGOs located within two of the study districts (Table 1). The mean age of the DHMs participating in the study was around 41 years, and the average time spent in their current management position was approximately three and half years. Six of the DHMs were female (40%), which was a bit less than the actual proportion (~50%) of females within the DHMTs. One of the regional participants and one of the NGO staff representatives were females. No systematic differences were observed between men and women in the interviews, and the results do thus not emphasize gender related perspectives.

**Table 1:** Overview of interview participants.

**Occupation**	***n***
Regional level	**4**
Regional Director	1
Research Officer	1
Administrator	1
Human Resource Officer	1
District level	**15**
District Director of Health Services	3
Deputy Director of Nursing Services	1
Health Administrator	1
Health Information Officer	4
Nutrition Officer	2
Disease Control Officer	2
Public Health Nurse	1
Staff from health-related non-governmental organizations within the districts	**2**
**Total**	**21**

The sample represented the majority of core DHMT positions within the selected districts. However, we were unable to interview a health promotion, human resource and supply officer due to them being occupied by the ongoing national mass distribution of bed nets.

Contextual, organizational and individual aspects, and how they relate and interrelate to shape management at district level, are discussed, respectively, in the sections below.

### Contextual Aspects

Study participants at district, regional and NGO level, repeatedly mentioned the infrastructure and physical environment surrounding the district health facilities as a barrier for the DHMs to carry out their activities, in particular those posted in rural terrains. Not only did they report on the DHMs difficulties in getting to the facilities for their essential supervision and monitoring responsibilities, but they also expressed concerns about the distance they had to travel to work at the DHA.

“*We do not have accommodation around, we are all living far away, so we cannot come easily to the office to do some work. People also come late because of that reason“* (DHM).

Appropriate accommodation is rarely offered to the DHMs when they are posted to a position in the district. This poses a challenge because the number of effective work hours are reduced significantly as they have to travel long distances to get to and from work. Moreover, in a setting with poor infrastructure, this poses a substantial safety risk at each commute. One manager mentioned how his colleague had been involved in an accident and lost her ability to walk. The limited number of motor vehicles, as well as money for fuel, were also mentioned by study participants as a hurdle for the DHMs in terms of carrying out their essential monitoring and supervision activities.

The inadequate resources allocated to the districts not only manifests in mobility issues and thus activities that cannot be carried out due to long commuting distances and time spent on traveling, it further manifest in work places that diminish effectivity and efficiency, i.e., poor lighting, lack of internet connection, hot office temperatures, overcrowded offices and lack of equipment at health facilities.

“*…look at this office…this is where we work. (…) It does not really depict a well-performing office. When it comes to performance the space that you will be allocated with will add on to your performance. Your office should not be as such when you enter the office and within 5–10 minutes you are tired”* (DHM).

The regional adminstrators also emphasized these challenges, and demonstrated frustration about their inability to address the issues at district level, due to delayed release of district health funds and health insurance reimbursement from the national level.

“*…if the health insurance is not giving back (…) then how do we get the money to buy the resources and things. That is the issue. So part of the problem is the payment of the services that we have rendered to health insurance which is not being paid back. So resources are locked*.” (RHA).

The shortage of human resources for health, both at the frontline and within the DHAs, and the DHMs limited decision-space to hire additional staff were emphasized by DHMs, as well as by RHAs and NGO staff members. The shortage within the DHA, forces the DHMs to take on responsibilities that they are not equipped or prepared for, and increases their workload. The overburden may render them inefficient in their core duties. The quote below demonstrates how a DHM took on myriad roles during his previous posting

“*When I was in my old place, I was a transport officer, a CHPS coordinator, I was the Procurement Officer and the Nutrition Officer at the same time. And any time the Disease Control Officer wasn't there I acted as the Disease Control Officer”* (DHM).

Meanwhile, the staff shortage at the frontlines, hinder DHMTs in their planning, coordination and execution of activities.

“*because they* [HR at sub-district level] *are not there, it means that all these tasks not actually are performed for the sub-district (…) meaning the system can only be weak because the pillars are not working”* (DHM).

The regional health administrators and the DDHS stated that annual objectives frequently were unmet due to the lack of financial, material and human resources. Moreover, one of the NGO respondents stated that she had experienced that DHMs due to a lack of resources and delayed funding were restrained from carrying out monitoring activities, and for example following up on frontline health workers that the NGO had supported the DHMT in training, causing the trainees to lose their newly acquired skills.

Study participants from the region, districts and NGOs, emphasized the importance of the DHMs having a good relationship with the officials at the district assembly in order for them to receive additional resources and support to achieve district health objectives.

“*(…) we are all competing for the same resources. Education, health, agriculture (…). We are competing, so we need to have a very workable relationship with them* [the District Assembly]” (DHM).

Presence of NGOs within the districts and a good working relationship between the DHMTs and these, as well as with donor partners play a role in DHMTs reaching their goals of improving health indicators. Not only because the NGOs may support the DHMTs with material resources and capacity building at the sub-district level, but also because the NGOs independently work on projects that aim to improve health outcomes within the district. Non-governmental organizations were not present within all the study districts.

“*(…) when an NGO comes in and tells me that their objective is to reduce maternal or neonatal deaths at least that burden will be off my chest. They will be coming to help me to surmount those problems.”* (DHM).

### Organizational Aspects

At the organizational level, structures and processes supporting the DHMTs in carrying out their tasks, were identified. The ones mostly emphasized as enhancing the capacity of the DHMs include (1) positive team dynamics, frequent information and knowledge sharing within the DHMTs via Whatsapp groups and weekly meetings; and (2) a good and highly dynamic relationship between the district and regional level with regular communication and frequent supervisory check-ins. Moreover, national directives (i.e., work plans, job descriptions, policies and guidelines) and the availability of health and HR information systems for surveillance, were mentioned as supporting the DHMs in carrying out their duties. However, DHMs raised concerns about the information systems relying on poor connection, as well as on substandard data collection and reporting at sub-district levels. Moreover, job descriptions were not always aligning with actual responsibilities and data reporting forms were frequently outdated. In addition, multiple DHMs expressed a need for additional directives (i.e., on community engagement, social and resource mobilization).

Another emphasized management support structure was the performance management system in which structured appraisals take place, both between the DDHS and RHA, as well as between the DDHS and the remaining DHMs. The system provides the DDHS and DHMs with supportive supervision, feedback and holds them accountable. Nevertheless, the system was undermined by the fact that identified strengths and needs rarely could be rewarded or addressed due to a lack of institutional support. The RHA and some DHMTs had established informal award systems, for example best DHMT and employee prizes, respectively, however this form of recognition was generally not described by the DHMs as effective in terms of motivating them to do a better job.

“*Formally the district is supposed to sponsor some people to go and do some management training. But anytime those opportunities come, they tell us they do not have the funds to be able to support. So when you have a chance to do, for instance, when I went and did my management training I sponsored it myself.”* (DHM).

Both DHMs and regional administrators reported that DHMs agendas were dominated by vertical programs. These were perceived positively in terms of adding resources to the district health system, however were described as contributing to a culture with little focus on systems strengthening. The vertical programs often have rigid requirements, inflexible funding, and come along with separate reporting systems that are not integrated into existing systems. The DHMs have limited authority to decide on how to utilize the resources, and are hindered in integrating them in a way that strengthens the overall health system.

“*…the vertical programs come with spreadsheets that have direct and very fixed, rigid requirements (…). The assumption of the program funders is that the* [existing district health] *system is working. (…). If the funding of a vertical program was flexible to integration, in that case, it can be also integrated in a way that will make both the system and the vertical programs sustainable.”* (DHM).

Continuous community engagement was for example highlighted by a DDHS as being critical for sustaining health improvements within the districts, however the lack of flexible resources and guiding policies, restrained them from carrying out such activities. Moreover, a DHM stated that the prescriptive directives from higher levels contributed to an organizational culture that not encourages DHMs to make data-driven decisions, as demonstrated by the quote below

“*We do not make decisions out of data (…) I have been trained to use data. But we could have an interaction, and most of the information I am giving you will not be based on the knowledge that I have. Why? Because we do not have this culture* [to use data]. *(…) you have to influence by leadership (…) for the culture to change and become more adaptive to what the data shows”* (DHM).

The vertical programs frequently target “popular” areas, such as disease control, leaving the DHMs with limited funds to cover other areas. To make the most of the allocated resources, integrated monitoring takes place, in which DHMs go jointly to the sub-districts and monitor a wide range of service areas. This ensures efficient use of scarce resources by giving “neglected” areas some attention. However, it reportedly also compromises the overall quality of the essential monitoring and supervision activities.

“*…you don't get time to do a detailed monitoring. (…). When you're doing the monitoring and support visit you are supposed to sit there and watch the person do the work, and if there are loop holes or some problems you take over and let the person observe you, so that you can correct them”* (DHM).

Nevertheless, the DHMs stated that the integrated monitoring provides them with a common understanding of the different DHMT members' role and responsibilities, which is helpful in situations where a DHM is transferred or on leave, and others have to take over.

### Aspects at the Individual Level

As depicted at the individual level in the framework (Figure 1), effective and efficient DHMTs are likely to be determined by: (1) the DHMs perception of their role; (2) their abilities to carry out their responsibilities; and ultimately, by (3) the effort they put into carrying out their tasks.

The DHMs appeared to have a clear perception of their role due to the dynamics and frequent information sharing within the DHMTs, and the strong relationship with the regional level who provides supportive supervision. However, the shortage of HR, which causes the DHMs to take on additional roles, imposed a risk of them taking on roles they were not prepared sufficiently for.

The DHMs ability to meet objectives depends on their competencies, as well as on whether they have autonomy to make decisions and resources to execute plans. As described in the sections above, the two latter aspects were limited. In terms of competencies, the study participants reported that the DDHS' are required to have a Master's degree in Public Health and some years of public health experience. However, requirements of leadership or managerial experience were not formalized. This was a concern at the regional level.

“*The leaders who are appointed… sometimes have no leadership skills… in terms of cannot convince…cannot lead meetings…cannot hold any stakeholder meetings with communities and with the persons working there. So it's a gap. So even if you are given the best of resources, you can't perform. So the structures, as getting people who are qualified, and then making sure roles are specified…”* (RHA).

Nevertheless, the regional health administrators simultaneously stated that the 26 DDHS' within the Eastern Region at the time of this study were well-qualified. They all possessed the required qualifications, in addition to having a certificate in Health Administration and Management from the Ghana Institute of Management and Public Administration (GIMPA). This certificate is recommended by the national level, yet the DHMTs have to find place to fund it within the given district health budget. In addition, DDHS candidates with no managerial experience are recommended to understudy a practicing DDHS for some months prior to taking the position. Meanwhile, there appeared to be no formalized requirements for the remaining DHMs.

“*…the appointment of these managers at the district and sub-district level should be formalized. Like they appoint DDHS. Some laid down criteria will be there, so that the person qualify”* (RHA).

The majority of the DHMs reported not having received any formal management and leadership training prior to being assigned to their role, but having acquired their competencies at their job. Nevertheless, general management and leadership skills among all DHMs, and not only the DDHS, were called for by the regional administrators, NGOs as well as by the DHMs themselves. Leadership skills in particular were described as being critical for DHMs to motivate frontline staff within the limited resource settings, and to compete with other sectors on resource inputs from the district assembly and NGOs, who frequently serve several sectors within the districts. To enhance the DHMs general management and leadership skills, the district has to fund their training from its own funds. This combined with no one to cover for the person who goes away for training, results in such trainings rarely being offered to the DHMs. Meanwhile, trainings provided through vertical programs were described as primarily targeting the DHMs technical skills rather than their general management and leadership skills.

Lastly, the effort the DHMTs put into carrying out their activities were positively affected by the DHMT dynamics and supportive supervision from the regional level and respective DDHS'. Nevertheless, various aspects also appeared to impede their motivation, including the limited resources and opportunities for professional growth, as well as their restricted autonomy to make decisions.

“*…you have highly trained leaders* [referring to the DDHS']*, but because of the way they* [higher levels] *are managing the system, they are dampening their spirit (…) So though you can do a lot, you have the skills to perform…but the way things are done we are unable to do anything.”* (DHM).

The sections above and the quote below demonstrate how contextual and organizational aspects influence the individual practices of DHMs, and that these need to be taken into consideration when aiming to strengthen district health management.

“*You can train the person, but no matter how you train the person (…) if there is a system failure…you can have all the competent people in the system because (…) you are not going to work in isolation. (…). You are looking at management as a system (…) you need to make sure that all the systems are working properly (…)”* (RHA).

## Discussion

The aim of this study was to explore how district health management emerges, hereunder the contextual, organizational and individual aspects that enable or hinder DHMs in carrying out their functions. Our findings paint a picture of district health management in which it is evident that management capacity not emerges from the competencies of the individual managers alone, but through a complex interplay of elements within the different levels. The political, social and economic context, such as human, material and financial resource availability, mode of decentralization in terms of decision-making, and priorities among elected officials and other health partners, affect organizational structures, processes and values, which in turn affect the DHMs abilities and motivation to carry out their duties.

Supported by previous studies, this study identified contextual aspects, such as delayed release of district health funds; poor infrastructure and working conditions; staff shortages at district and sub-district level; good working relationships within district and between districts and regions; lack of opportunities for professional development; and limited decision-making power, as affecting the DHMs ability and willingness to carry out their tasks effectively and efficiently (13, 25, 43–46). Moreover, management and in particular leadership competencies among DHMs, including abilities to effectively communicate, inspire and align employees and potential partners that can provide support, has in this study, as well as in other studies, been emphasized as a mean to achieve results within the given contextual and organizational arrangements (47–49).

### Insights for Strengthening Management at District Level

Findings of this study underline that strengthening management capacity at district level should be considered at multiple levels rather than only at the individual level. The contextual level affects the organizational level through its provision of incentives and an enabling environment (50–53). The organizational level affects the individuals' ability and willingness to perform by providing a framework of structures, processes and procedures (11). In turn, the individual practices at the district level take part in maintaining and shaping the organizational context. The contextual and organizational aspects are less tangible to change compared with individual competencies, however critical for creating sustainable improvements (16, 29).

Certain policies need to be in place in order to ensure an enabling and incentivizing environment for the DHMTs. Policies and accountability structures should ensure that resource inputs are distributed in a timely manner. Similar to other studies in Ghana, we found that untimely release of national health insurance reimbursements, as well as delays in state and development partner funds, demoralize and prevent DHMs from carrying out their activities (25, 33, 54). We did not explore the bottlenecks in the disbursement of funds, however these must be identified and addressed to ensure that the DHMs have the necessary resources to carry out their tasks. Advocating for enhancing the commitment and budgetary allocation to health by the Government of Ghana may also serve as a mean to create a more enabling environment for DHMs. Currently the allocation to health falls short of the 15% pledge in the Abuja declaration (55). Moreover, in concordance with another study from Ghana, we found that resource inputs from local governments to some extent rely on personality driven relations and preferences among elected officials at the district assemblies (13). Currently, the Local Government Act 462 does not clearly define the roles and responsibilities of district assemblies in terms of health, and practices relying on the DHMs ability to lobby are thus encouraged.

The organizational context is largely affected by political decisions on health system organization, and the scarcity of resources in the broader context. The hierarchical, top-down approach to planning and problem-solving may hinder creative and adaptive district management that are responsive to local health challenges (13, 56, 57).

This study demonstrated that prescriptive directives and fixed funding diminish bottom-up learning, data-driven decision-making, and limits the DHMs from integrating funds into activities, such as community engagement that strengthens the district health system in ways that are necessary for sustaining improvements. In turn, this lessens the DHMs sense of ownership and internalization of organizational goals and thus the effort they execute (13, 51, 52).

Other studies have also emphasized that vertical and donor programs in Ghana may interrupt and delay coordinated activities at the district level, as they are poorly planned, communicated, and come along with separate reporting systems (3, 25, 58). The latter was confirmed in this study. Lack of integrated health information systems has been associated with poor quality data (58). Thus, parallel reporting systems should be prevented. Ways to do so may be explored in future research or by looking to other countries, such as Rwanda where the MoH has commenced implementation of a nationwide comprehensive electronic medical record system that ensures that parallel systems are not created (58). Furthermore, in order for DHMs to make decisions based on reliable and timely information, our findings indicate that information systems firstly must be optimized by strengthening data reporting mechanisms at sub-district level and by ensuring access to information systems in all geographic areas, including those with poor tele network.

Adaptations in organizational structures and processes are needed to enable DHMs to strengthen district health systems. Nonetheless, these have evolved through decades, and will take time and political will to change (29). Some studies suggest that a critical mass of people with leadership skills at district level is likely to push changes in the context (48, 59). In addition, learning from leadership development history in Ghana, confident and capacitated DHMTs may push for more decision-space, as well as for changes in current organizational structures and cultures that diminish bottom-up learning (35). In addition, they may hold higher levels accountable to delayed disbursements and to enhancing district health funding.

Ensuring a sustained critical mass of leaders that are able and willing to push for change, firstly require policies that ensure management and leadership competencies among assigned health system managers. This study demonstrated that all DDHS' within the Eastern Region held a certificate in Health Administration and Management, largely due to this being an institutionalized recommendation for filling this role. Meanwhile, there were no formalized management and leadership requirements for the remaining DHMs, similar to in other LMICs (47). Consequently, these had little exposure to management and leadership training, and district health funds were not prioritized to enhance these competencies.

Moreover, sustained management capacity at district level may be enhanced by building on existing structures and capacities. Confirmed by another study in Ghana, we identified aspects that enable DHMs to carry out their functions, including good relations within and between the districts and regions; as well as the performance management system, in which supportive supervision takes place (13). The regions who have positive relations with the districts are well-positioned to match learning between well-performing and less performing districts based on insights from the performance management system. Peer-review of management practices in which DHMTs learn from other DHMTs take place to some extent in the Eastern Region of Ghana. However, it has to our knowledge not been institutionalized and is not widely applied, demonstrated by neither of the DHMs in current study emphasizing peer-learning from other districts. A study from the Eastern Region of Ghana found that better performing districts had transformational leaders that use a participatory approach that promotes bottom-up communication to solve problems (60). Inter-district learning may be an advantageous and sustainable approach to strengthen management and leadership as peers are familiar with the contextual barriers, and how to achieve better performance with the means available within the given culture and context (61). Moreover, it is inexpensive, and learning from the LDP, using peers for mentorship and coaching may enhance commitment among the DHMs, compared with a top-down approach. Future research may look into approaches to strengthen management and leadership that build on structures and capacities that already are in place.

### Strengths and Limitations

To understand situations systemically means to put them into context, and a “system approach” to capacity strengthening at district level, thus first and foremost requires a thorough understanding of the context in which the DHMs operate (51). We explored this context through the eyes of DHMs and those they work closely with. The DHMs are at the center of management strengthening interventions, yet their voices are often left unheard despite knowing best what capacity is needed and how it best can be developed and sustained within their culture and context (61). Moreover, by including individuals who work closely with the DHMs, we gained a richer and more objective view of aspects influencing district health management. To gain a further understanding of the context in which the DHMs are embedded, future research may however also include other district health actors, including local governments, sub-district health teams and frontline health workers.

Certain aspects affecting how district health management emerges may have been overseen as this study not included all core members of the DHMTs due to the ongoing mass distribution of bed nets. However, we argue that the risk of having overseen any major challenges is minor as multiple DDHS' and regional health administrators who have a holistic overview were included in the study.

Data was categorized into contextual, organizational and individual factors that may impact how district health management emerges, however these systemic layers and categorizations of elements within these, are not definitive, and may leave out some factors that affect management capacity. Other studies have for example emphasized that DHMs also are influenced by their personal family and socio-economic situation, remuneration, and stability of employment (62).

## Conclusion

This study draws attention to aspects at the individual, organizational, and contextual level that influence how district health management emerges. Aspects that enable and hinder DHMs in carrying out their functions were identified, and may provide national policy-makers, donors, and researchers with a deeper understanding of factors that should be taken into consideration when developing, planning, implementing, and assessing capacity-building strategies targeted at strengthening district health management.

## Data Availability Statement

The datasets presented in this article are not readily available because it contains information that could compromise the privacy of study participants. Requests to access the datasets should be directed to annechristine.heerdegen@swisstph.ch.

## Ethics Statement

Ethical clearance was obtained by the Research Ethics Committee of Liverpool School of Tropical Medicine (ID No. 17–046) and the Ghana Health Service Ethics Review Committee (No. GHS-ERC004/01/20). The study participants provided their written informed consent to participate in this study.

## Author Contributions

AH: design, data acquisition, analysis, interpretation, and drafting. JG: design, interpretation, and critical revision. SA, SAA, and MA: data acquisition and critical revision. KW: design and critical revision. All authors have contributed to the article and approved the submitted version.

## Acknowledgements

This study is an output from the PERFORM2Scale project (2017–2021): Strengthening management at district level to support the achievement of Universal Health Coverage, funded by the European Commission (reference number: 733360). The project involves a consortium of seven partners: Liverpool School of Tropical Medicine, United Kingdom; Trinity College and Maynooth University, Ireland; Royal Tropical Institute Amsterdam, Netherlands; School of Public Health, University of Ghana, Ghana; Swiss Tropical and Public Health Institute, Switzerland; REACH Trust, Malawi; and School of Public Health, Makerere University, Uganda. The authors would like to acknowledge and thank all the study participants who took their precious time to talk with us and inform the findings of this study.

## Conflict of Interest

The authors declare that the research was conducted in the absence of any commercial or financial relationships that could be construed as a potential conflict of interest.
